# The Contribution of Environmental Science to Mental Health Research: A Scoping Review

**DOI:** 10.3390/ijerph20075278

**Published:** 2023-03-27

**Authors:** Michaela Roberts, Kathryn Colley, Margaret Currie, Antonia Eastwood, Kuang-Heng Li, Lisa M. Avery, Lindsay C. Beevers, Isobel Braithwaite, Martin Dallimer, Zoe G. Davies, Helen L. Fisher, Christopher J. Gidlow, Anjum Memon, Ian S. Mudway, Larissa A. Naylor, Stefan Reis, Pete Smith, Stephen A. Stansfeld, Stephanie Wilkie, Katherine N. Irvine

**Affiliations:** 1 Social, Economic and Geographical Sciences Department, The James Hutton Institute, Craigiebuckler, Aberdeen, Scotland AB15 8QH, UK; 2 Environmental and Biochemical Sciences Department, The James Hutton Institute, Craigiebuckler, Aberdeen, Scotland AB15 8QH, UK; 3 Institute of Infrastructure and Environment, School of Energy, Geoscience, Infrastructure and Society, Heriot-Watt University, Edinburgh EH14 4AS, UK; 4 UCL Institute of Health Informatics, 222 Euston Road, London NW1 2DA, UK; 5 Sustainability Research Institute, School of Earth and Environment, University of Leeds, Leeds LS2 9JT, UK; 6 Durrell Institute of Conservation and Ecology (DICE), School of Anthropology and Conservation, University of Kent, Canterbury, Kent CT2 7NR, UK; 7 King’s College London, Social Genetic and Developmental Psychiatry Centre, Institute of Psychiatry, Psychology & Neuroscience, 16 De Crespigny Park, London SE5 8AF, UK; 8 Economic & Social Research Council (ESRC) Centre for Society and Mental Health, King’s College London, 44-46 Aldwych, London WC2B 4LL, UK; 9 Centre for Health and Development (CHAD), Staffordshire University, Leek Road, Stoke-on-Trent ST4 2DF, UK; 10 Department of Primary Care and Public Health, Brighton and Sussex Medical School, Brighton BN1 9PH, UK; 11 MRC Centre for Environment and Health, Imperial College London, White City Campus, London W12 0BZ, UK; 12 NIHR Health Protection Research Units in Environmental Exposures and Health, and Chemical and Radiation Threats and Hazards, Imperial College London, White City Campus, London W12 0BZ, UK; 13 School of Geographical & Earth Sciences, East Quadrangle, University of Glasgow, Glasgow G12 8QQ, UK; 14 UK Centre for Ecology & Hydrology, Bush Estate, Penicuik EH26 0QB, UK; 15 European Centre for Environment and Human Health, University of Exeter Medical School, Knowledge Spa, Truro, Cornwall TR1 3HD, UK; 16 Institute of Biological and Environmental Sciences, University of Aberdeen, 23 St Machar Drive, Aberdeen AB24 3UU, UK; 17 Centre for Psychiatry, Barts and the London School of Medicine, Queen Mary University of London, Charterhouse Square, London EC1M 6BQ, UK; 18 School of Psychology, Murray Library, City Campus, University of Sunderland, Sunderland SR1 3SD, UK

**Keywords:** mental wellbeing, cognitive development, cognitive decline, environmental epidemiology, physical environment, chemical environment, biological environment

## Abstract

Mental health is influenced by multiple complex and interacting genetic, psychological, social, and environmental factors. As such, developing state-of-the-art mental health knowledge requires collaboration across academic disciplines, including environmental science. To assess the current contribution of environmental science to this field, a scoping review of the literature on environmental influences on mental health (including conditions of cognitive development and decline) was conducted. The review protocol was developed in consultation with experts working across mental health and environmental science. The scoping review included 202 English-language papers, published between 2010 and 2020 (prior to the COVID-19 pandemic), on environmental themes that had not already been the subject of recent systematic reviews; 26 reviews on climate change, flooding, air pollution, and urban green space were additionally considered. Studies largely focused on populations in the USA, China, or Europe and involved limited environmental science input. Environmental science research methods are primarily focused on quantitative approaches utilising secondary datasets or field data. Mental health measurement was dominated by the use of self-report psychometric scales. Measures of environmental states or exposures were often lacking in specificity (e.g., limited to the presence or absence of an environmental state). Based on the scoping review findings and our synthesis of the recent reviews, a research agenda for environmental science’s future contribution to mental health scholarship is set out. This includes recommendations to expand the geographical scope and broaden the representation of different environmental science areas, improve measurement of environmental exposure, prioritise experimental and longitudinal research designs, and giving greater consideration to variation between and within communities and the mediating pathways by which environment influences mental health. There is also considerable opportunity to increase interdisciplinarity within the field via the integration of conceptual models, the inclusion of mixed methods and qualitative approaches, as well as further consideration of the socio-political context and the environmental states that can help support good mental health. The findings were used to propose a conceptual model to parse contributions and connections between environmental science and mental health to inform future studies.

## 1. Introduction

Issues surrounding mental health and wellbeing are one of the major international public health challenges of our time. It represents a “wicked problem” that necessitates interdisciplinary collaboration across the biomedical, social, economic, and natural sciences [1]. Prior to the COVID-19 global pandemic, around 16% of the world’s population was estimated to be affected by mental or addictive disorders [2]. In Europe, the prevalence of mental health disorders increased by around 16% between 2005 and 2015 [3]. Concurrently, there has been growing international interest in the role individuals, communities, and societies play in fostering positive mental health and wellbeing, as exemplified by the rise of “happiness economics” as a counterpoint to the reliance on traditional metrics such as gross domestic product as a basis for public policy [4].

Building a fuller understanding of the impact of the environmental context on mental health has become a priority for the global mental health research agenda [5,6]. Numerous recent reviews evidence the importance of the environment for mental health through impacts associated with, for example, climate change [7] and related flooding events [8,9], air pollution [10] and access to urban green space [11,12,13]. In the social sciences, this impact is often explored via frameworks that consider both the nature experience and nature exposure pathways linking environments with health and wellbeing [14,15]. The interplay between an individual’s subjective experience of nature and their physiological exposure to environmental factors such as air pollutants or positive microbiomes has led to growing recognition of the myriad environmental drivers of mental health and wellbeing, all of which point to a significant potential role for environmental science to contribute to the mental health research agenda. However, our understanding of the current role of environmental *science*, as opposed to environmental factors or determinants, in mental health research is limited. There is a need for clear agenda-setting for environmental science’s future contribution to mental health scholarship.

## 2. Review Aim, Objectives and Research Questions (Step 1)

The aim of this scoping review was to improve our understanding of environmental science’s role in mental health research, including mental disorders, positive wellbeing, and conditions relating to cognitive development and decline (see Box 1 for definitions). The objectives were to: map the existing literature addressing environmental influences on mental health; assess the extent and form of environmental science’s contribution; and provide recommendations for both mental health- and environmental science-allied professions to highlight how they may benefit from each other to further understanding of the environment-mental health connection. A detailed review of environmental impacts on specific mental health outcomes through a review of the results was beyond the scope of this review because such impacts are better reviewed at a finer scale (e.g., a specific region, environment, or mental health condition) and with different review methodologies (e.g., systematic reviews).

To achieve the aim and objectives, four questions guided the scoping review:

1. What is the current contribution of environmental science to mental health research? This includes consideration of the pathways by which the environment impacts mental health and wellbeing, including conditions of cognitive development and decline, and how environmental science has been leveraged to understand these pathways or impacts;
2. What are the current research designs and methodological approaches being used in environmental science and mental health research?
3. How does the relationship between environmental science and mental health research relate to existing evidence linking mental health and wellbeing to demographic, social, economic, and genetic determinants?
4. What are the evidence gaps and opportunities for the contribution of environmental science to mental health research?

Box 1 **Concepts and definitions used for the scoping review.**

**
Mental health
**

The World Health Organization (WHO) defines health as “a state of complete physical, mental and social wellbeing and not merely the absence of disease or infirmity” [16]. In this paper, mental health is therefore conceptualised as incorporating aspects of positive wellbeing (see below) as well as the presence or absence of a mental illness or disorder. We also expand the scope of mental health, for the purposes of the review, to encompass conditions relating to cognitive development (e.g., autism, attention deficit hyperactivity disorder) and decline (e.g., dementia) following, e.g., [5].

**
Wellbeing
**

Wellbeing is conceptualised here as a subjectively experienced positive mental state consisting of two components, hedonic (i.e., pleasure, enjoyment) and eudaimonic (i.e., purpose in life, personal growth) [17,18,19]. Definitions of wellbeing focused on the objective indicators of quality of life, such as income (e.g., [20]), are excluded. Wellbeing is differentiated from mood; mood pertains to short-lived and fluctuating affective states, whereas wellbeing is assessed as a global state or aggregate of affective states over a given period of time (e.g., a week, a month) [21].

**
Environmental science
**

This refers to scientific fields focusing on the study of physical, chemical, and biological processes in the natural environment [22]. The term includes (but is not restricted to) disciplines such as ecology, geology, physical geography, hydrology, geomorphology, plant science, soil science, zoology, environmental chemistry, oceanography, meteorology, and climatology.

**
Determinants of (mental) health
**

In referring to determinants of health, we consider the broad range of biological (including genetic), psychological, social, and environmental factors that may influence human health, drawing on a number of conceptual models of health [23,24,25,26]. In relation to the role of environmental science, the natural environment (comprising air, water, land, and habitats) and the global ecosystem (incorporating climate change and biodiversity) determinants, as highlighted in Barton and Grant’s [25] Health Map, are our focus in this paper.

**Pathways of impact**

We conceptualise the pathways by which the environment impacts mental health according to the DPSEEA (Drivers, Pressures, State, Exposure, Effect, and Actions) framework ([27], modified by [28,29] to include experiences). This framework elaborates a causal chain by which an environmental *state* (e.g., natural resources, natural hazards, pollution) results in an *effect* on health (in terms of wellbeing, morbidity, or mortality) via *exposure* or *experience* by humans occurring within a wider social, economic, and environmental *context*.

## 3. Methods

### 3.1. Overview

Varied evidence review methodologies exist, each with distinct aims and guidance [30]. According to Munn and colleagues [31], these can range from systematic reviews, a process for reviewing and appraising the evidence based on a limited, focused question, to scoping reviews, which instead aim to address broad research question(s) with the purpose of consolidating the evidence to determine types of available evidence, methodological/conceptual trends, and knowledge gaps that should be addressed in future studies, particularly where the topic is one that is interdisciplinary. As such, a scoping review was the appropriate methodology to achieve the aims and objectives of this study [32]. Scoping reviews do not include assessment of the quality of the evidence or risk of bias in published findings [33,34], which are relevant to systematic reviews and are outside the parameters of this review.

Scoping reviews follow a rigorous 6-step process [32,35,36,37]. Step 1 involves identifying the research question(s) and was presented in the introduction. Steps 2 (identifying relevant studies), 3 (study selection), and 4 (charting the data) are presented in this section. Step 5 (collating, summarizing, and reporting the results) follows in the results section, which also involved Step 6 (consultation with experts on the summary findings).

### 3.2. Identification of Relevant Studies (Step 2)

#### 3.2.1. Protocol

A scoping review protocol was developed with input from experts in mental health and environmental science drawn from research, policy, and practice [32,33,35,37]. For the full protocol, refer to the Supplementary Materials. Briefly, the protocol details the procedures used to specify research questions (Step 1 detailed above) and the identification of data sources, study selection and inclusion criteria, and data charting (Steps 2–4).

#### 3.2.2. Inclusion/Exclusion Criteria

English-language peer-reviewed and grey literature were reviewed. Publication dates were limited to between January 2010 and August 2020 to capture current research directions up to the emergence of the COVID-19 pandemic (allowing for some lag time in publication). To ensure a focus on human mental health, only studies conducted with human participants were included; those reporting non-human findings were excluded.

#### 3.2.3. Information Sources and Search Strategy

Searches for the relevant literature were carried out in the Web of Science (all databases, selected for the breadth of publications across the social sciences), PubMed (to target mental health-specific publications), and the British Library (to identify grey literature). To identify government reports, the research portals of the European Union (EU), the United Kingdom (UK), and the devolved governments of England, Scotland, Wales, and Northern Ireland were also searched for the term “mental health”. Environmental science was considered in terms of physical, chemical, and biological processes in the natural environment ([22]; Box 1). The WHO’s [16] definition of mental health was used (see Box 1). An initial set of keywords drawing on the various environmental domains (air, water, land, habitats, biodiversity, climate change) that influence health were developed by the project team (MR, KC, MC, AE, KNI). This process was guided by Barton and Grant’s [25] Health Map of the wider determinants of human health. The initial set of keywords was validated and refined in a series of interdisciplinary workshops with experts from mental health and environmental science disciplines and further refined through preliminary searches on the Web of Science (see Supplementary Materials for full details). Keywords (Table 1) were searched in the title, abstract, and author-assigned keywords in the Web of Science (on 10 August 2020), in the title and abstract in PubMed (on 11 August 2020), and in the British Library (on 13 August 2020). We also searched the EU, UK, and devolved government research portals for the term “mental health” (13 August 2020).

### 3.3. Study Selection (Step 3)

Paper titles were screened by a single researcher (MR). Study selection began with the removal of duplicate papers. Papers that did not include mental health or environmental science, were non-human animal studies, or were review, opinion, or descriptive papers were excluded at this stage. To ensure papers were not excluded where they may fit the review remit, a random sample of 10% of the titles were independently screened by a second reviewer (MC). Agreement between reviewers was tested through the Kappa statistic, with a score of 0.92 (confidence interval 0.84–0.99, 96% agreement). This indicates a near-perfect agreement on the acceptance of papers [38]. Papers that were not agreed upon were retained and included in the next stage of screening. Given the near-perfect agreement on acceptance, duplicate screening was not carried out at any further stage.

The remaining papers were screened by the abstract (MR). The final assessment of papers for eligibility occurred during the charting process (Step 4), and any remaining ineligible papers were removed following discussion between the charting team (MR, KC, MC, KNI).

#### Review Papers

To avoid duplicating the work of previous reviews, at the abstract screening stage we further excluded papers reporting studies on topics sufficiently covered by “robust” reviews published up to August 2020. These recently published reviews were identified during the search stage but had been excluded from the main scoping review because they were not primary research studies. The review papers therefore follow the same inclusion criteria as the primary data papers, in addition to “robustness”. We considered a review “robust” if it reported a systematic search protocol and searched at least one scientific database and one source of the grey literature. A topic was deemed sufficiently covered (i.e., excluded from our current scoping review) if the combined reviews on the topic: (i) covered at least 10 years with the latest date being no earlier than 2017 (allowing for realistic publishing delay); (ii) had global geographic coverage; (iii) included the entire population (e.g., not only children); and (iv) covered multiple dimensions of mental health rather than a singular named condition. This resulted in primary studies that focused on the following topics being excluded from the main scoping review: climate change, flooding, air pollution, and urban green space. Subsequently, any insights into environmental science and mental health research and future research opportunities related to these four topics presented in the results and discussion are based on a separate charting of these robust reviews, not the individual papers contributing to them.

### 3.4. Data Charting and Synthesis (Step 4)

Data were charted and extracted by four authors (MR, KC, MC, KNI) and entered into a spreadsheet designed based on recommended guidance and organised to address the research questions (Table 2) (e.g., [35]). Empirical findings related to the effectiveness/impact of environmental factors on mental health outcomes were not extracted because the review’s aims and objectives were concerned with how environmental science was incorporated into mental health research and how these disciplines may benefit from each other. Thus, the focus was on furthering understandings of disciplinary connections rather than the impacts of the environment on mental health per se.

With regards to the first research question presented in Table 2 (*What is the current contribution of environmental science to mental health research?*) it is worthy to note that when considering the “interaction between environmental science and mental health” we used the four categories identified by Huutoniemi et al. [39]. These include and are defined as: (i) Composite multidisciplinary—expertise in different fields combined, but research is still modular, “outsourcing” of part of a research project to use methods from another discipline but still framed within a single discipline; (ii) Empirical interdisciplinarity—integration of empirical data from multiple disciplines to answer a question about the relationship between both disciplines; (iii) Methodological interdisciplinarity—combining and integrating methods to suit the interdisciplinary nature of the question; (iv) Theoretical interdisciplinarity—synthesis of concepts, models, or theories from multiple disciplines, forming an interdisciplinary theory.

The identified environmental science topics were grouped (MR) into broader themes of similar topics within studies (e.g., “natural disaster” included hurricanes and earthquakes). Included studies were further clustered (MR) by similar methods. For example, rainfall records and water pollution records became “secondary spatial data”, direct measurements of ozone and noise became “environmental measurement”. Mental health outcomes were grouped into the WHO’s International Statistical Classification of Diseases and Related Health Problems, 10th revision (ICD-10; [40]). This enabled the charted data to be summarised and for patterns to be identified both within and between environmental science themes and mental health areas.

#### Reviews

Reviews of studies on climate change, flooding, air pollution, and urban green space were charted and extracted separately after the synthesis of the scoping review papers. As such, data from the review papers were extracted specifically regarding each research question directly, utilising categories developed through the main scoping review (e.g., how the paper described mental health methods) and a narrative description. This approach recognises that review papers present data differently from those reporting primary results.

## 4. Results (Step 5) Including Expert Consultations (Step 6)

Results are presented beginning with overall search results and then by research question. The main body of the results refers to the results found from our scoping review, with further insights from the existing reviews on climate change, flooding, air pollution, and green space included at the end.

### 4.1. Search Results

**Figure 1:**
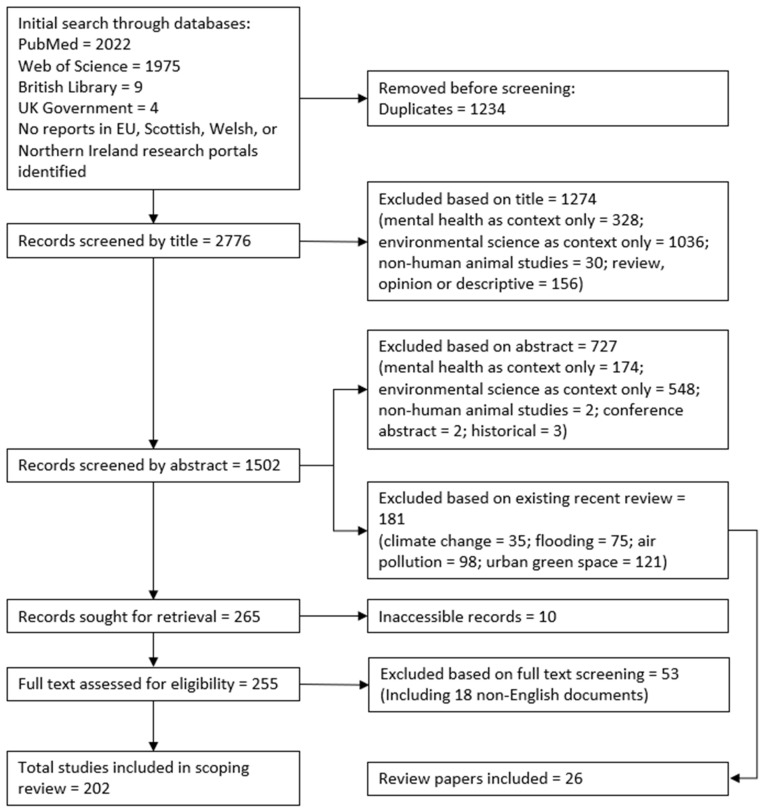
Screening record of scoping review and review papers. Review papers were excluded from the scoping review but retained for separate consideration where they concerned climate change, flooding, air pollution, and urban green space.

The flowchart in Figure 1 illustrates the screening process undertaken in the scoping review. From the initial 2776 unique papers identified, 202 were included in the final main scoping review. Twenty-six review papers were also considered on the topics of climate change, flooding, air pollution, and urban green space.

### 4.2. What Is the Current Contribution of Environmental Science to Mental Health Research?

Five core environmental science themes related to mental health research were identified: natural disasters, noise, chemical pollution, natural environments, and meteorological conditions (Table 3). There was high variability in the number of papers identified between themes. Within some themes, there was a dominance of one sub-theme (e.g., wildfires were the most prevalent type of natural disaster). We have reported on these sub-themes separately to prevent overshadowing the other papers within the core theme. Table 3 also incorporates the four review themes: climate change, flooding, air pollution, and urban green space.

**Figure 2:**
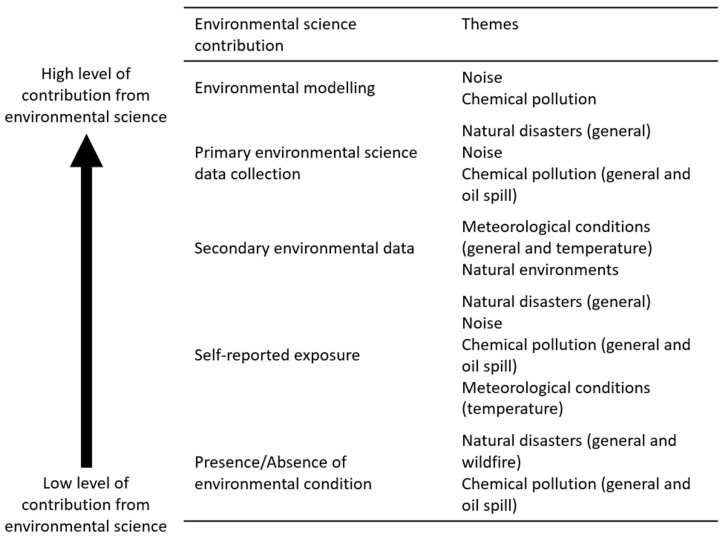
Varied levels of contributions of environmental science to mental health research. Arrow indicating the extent to which the environmental part of the study had contributions from environmental science.

The most basic contribution of environmental science to mental health research was the identification of either the presence or absence of an environmental state (Figure 2). This measurement of the presence of an environmental state itself, rather than an environmental exposure to that state, arguably better demonstrates a lack, rather than an involvement, of environmental science (i.e., the environment is simply present or not; it has not been further measured). This presumption of exposure based on the presence of an environmental state was the principal contribution of environmental science to research on natural disasters’ impacts on mental health and the only measure applied in the most common natural disaster considered, wildfire. Presumed exposure based on environmental state was also applied in chemical pollution research, including oil spills.

In natural disaster research, studies quantifying exposure predominantly used self-reported exposure with little environmental science input (Figure 2). Self-report measures were also used to assess noise [42,43,44,45,46,47,48,49], chemical pollution including oil spills [50,51,52,53,54,55,56,57], and temperature [58].

Several studies used secondary environmental data; these studies benefited from existing environmental science expertise despite the collection of environmental science data not being part of the study itself. Secondary data were used in all studies of the effects of meteorological conditions, including temperature, on mental health [59,60,61]. Secondary data were also used in measuring exposure to natural environments, assessing proximity to an environment type identified using land cover or land use classifications [62,63,64] (Figure 2).

There was a lack of primary environmental science data incorporated into studies. The few studies that did included drought impacts on mental health [65,66,67,68], in contrast to the other natural disaster topics that considered only the presence or absence of disaster. Another environmental measurement included was the impact of noise on mental health [42,43,69,70,71,72,73,74]. In pollution studies, chemical agent exposures were estimated, and oil spill characteristics have also been measured to quantify the extent of exposure. Exposures to pollen have additionally been examined in relation to mental health [75].

Mathematical modelling of environmental states was directly produced in only a few studies. These included studies modelling noise levels to estimate the impact on mental health [76,77,78,79,80] and another that modelled the exposure to chemical pollutants [81].

We categorised the mental health aspects of papers into those considering general mental health and wellbeing, those linked to mental health disorders classified in the ICD-10 [40], and those related to cognitive development or decline (Table 4). The highest number of papers considered general mental health. The most studied disorders were mood disorders (predominantly depression) and neurotic disorders (predominantly anxiety). Cognitive development and cognitive decline were the subjects of few studies (Table 4). Environmental science themes in relation to mental disorders were inconsistent, with disorders often only studied in relation to a small number of the themes (Table 5). Cognitive development and cognitive decline were most restricted in the themes within which they were studied, with papers on only noise and general pollution for development and natural disasters and meteorological conditions for decline (Table 5).

**Figure 3:**
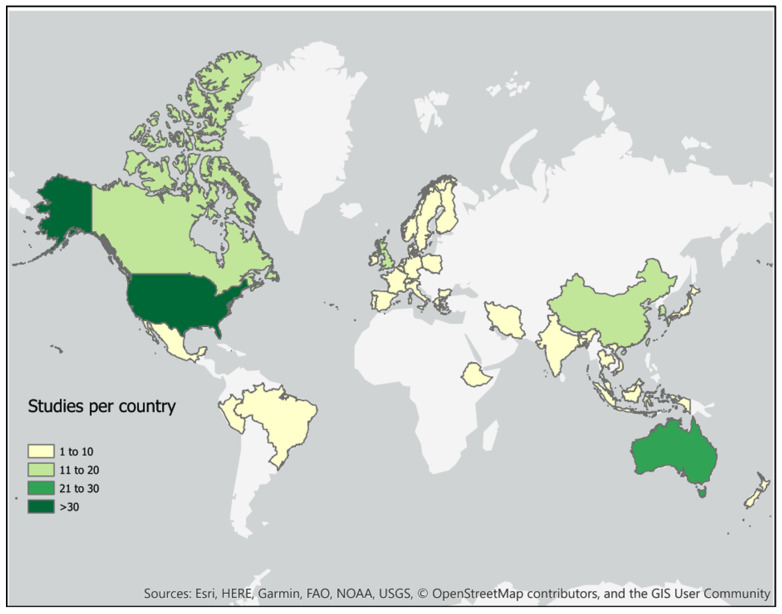
Study location by site of research. A single study may have sites in more than one location. This map does not include review papers.

The regional geographic distribution of the scoping review studies showed that these were mainly conducted in North America (predominantly the USA), Asia (predominantly China), or Europe (not limited to the EU) (Figure 3), though again there is variation across environmental science themes. The natural disasters papers had a wide geographic scope, though study sites in Africa were notably lacking. When looking specifically at wildfires, North American study sites dominated. Noise research has been focused on within Europe. Chemical pollutant studies cover both Asia and North America, with oil spill research dominated by studies investigating the Deep Horizon spill in the USA. Studies on natural environments were mainly situated in Europe. Meteorological conditions excluding temperature were studied mainly in North America and Asia, with temperature having broad geographic coverage.

#### 4.2.1. Evidence from Reviews (Climate Change, Flooding, Air Pollution, Urban Green Space)

Insight about the themes of climate change, flooding, air pollution, and urban green space was drawn from our synthesis of recently published reviews (Table 6). Studies with primary environmental data or created models of the environment were more common in research on air pollution than research on climate change or flooding. Urban green space research employed primary environmental data, but no modelling of the environment. The level of air pollution and the area of green space were often estimated directly, for example, through air quality sensors and land measurement, respectively. In contrast, research on climate change or flooding predominantly featured studies with data documenting the presence or absence of environmental conditions. Most reviews define climate change as a constellation of phenomena, including global warming, rising sea levels, and increased occurrences of extreme weather conditions, but the overall impact of climate change on mental health has not been quantified. The same is true for flooding. Most reviews did not attempt to produce a quantitative effect measure except for Braithwaite et al. [10], who included a meta-analysis of the effect of air pollution.

Reviews on the effect of climate change have mostly focused on general mental health and wellbeing, possibly due to a lack of available evidence (Table 6). Reviews on the effect of flooding were primarily concerned with PTSD and suicide, while reviews on the effect of air pollution paid special attention to cognitive function. The reviews adopted a broad geographic scope, although reviews discussing climate change and flooding were more likely to limit their discussion to environmental states in a particular country or region (Table 6).

### 4.3. What Are the Current Research Designs and Methodological Approaches Being Used in Environmental Science-Mental Health Research?

Most studies were approached from the perspective of the mental health field. Interaction between environmental science and mental health was largely empirical (as defined in [39]), with environmental science data incorporated alongside mental health data at an aggregate level but without further integrating methods. Studies were predominantly observational and considered the negative impacts of environmental factors on mental health. The exception was natural environments; these studies used interventions to examine the effect of forest activities [106,107]. The predominance of disaster literature may also explain the higher number of cross-sectional study designs, due to the scarcity of longitudinal data related to unpredictable natural events.

Studies mainly used field or secondary data. Human lab-based studies such as biomarker-based investigations were rare. Across all themes, a quantitative paradigm was primarily applied (Table 7), and regression models were the most common analysis technique. In those studies that used qualitative approaches, a range of methods were applied, including interviews and the review of documents using thematic analysis and grounded theory. Ethnographic, autoethnographic, and participatory research methods were also applied, though rarely.

The remainder of this section focuses on papers that used primary environmental data or created direct models of the environment as examples of where environmental science methods have been integrated with mental health research, thus demonstrating methodological interdisciplinarity [39].

Direct measurements of environmental variables were the most common method for environmental measurement and were found in drought, noise, and pollutant studies:

- In drought studies, rainfall data were collected alone [66,67] or in combination with: drought period [68], soil moisture [68], or water allocations [68]. These data could also be combined to create Hutchinson’s Drought Index [65,108,109].
- Noise monitoring was carried out using static noise monitors at the neighbourhood scale [69,71,72,73,74,78,110,111,112,113], recording peak and off-peak noise at varied locations to map noise across the study sites. Participants in one study carried portable noise sensors, enabling an individual noise map to be created [42].
- Pollutant studies used standard procedures, including for water [114,115,116], air [117], and chemical pollutants (e.g., arsenic, nitrate, uranium). Similar methods were used to measure oil spill characteristics to quantify the extent of exposure, including hazardous material, volume, surface area of the spill, magnitude, and dispersant volume [118]. Only studies of chemical pollutants used biomarkers indicative of exposure level, analysing parent compounds and their metabolites in hair and urine, to provide an estimate of the biological dose [53,114,119,120,121,122].

Direct modelling of the environment was limited. We found modelling only of noise, based on the physical forms of the environment and noise sources [76,77,78,79,80], and estimated exposure to chemical pollutants (tetrachloroethylene) through drinking water using historical maps along with leaching and transport models [81].

Regarding the mental health and wellbeing aspect of the identified research, there was little variation in methods between those used for general mental health, wellbeing, or identified disorders. To measure individual mental health (as opposed to population level), self-report scales were most common and included short-form health status questionnaires such as the General Health Questionnaire-12 (GHQ-12) or the Strengths and Difficulties Questionnaire, as well as author-developed scales. Self-report scales were also employed for considering mental health disorders, including post-traumatic stress disorder, anxiety and depression, substance abuse, and suicidal thoughts. Parental reports of children’s behaviour [43] or the Child Behaviour Checklist [123] have been used in studies of the impacts of noise. Less common methods for measuring individual level mental health included structured psychiatric interviews (noise: [71,124]; natural disasters: [50]), ethnographic [125] or qualitative (oil spills: [56]) interviews.

Administrative records have been employed in population-level studies, including hospital diagnoses, prescription data, and medical insurance data. Death records were included in studies of suicide rates (noise: [126,127]; pollution: [117], and economic estimations of disease burden have also been used in population-level studies [61,128].

Within the scoping review papers, one of the mental health research areas to which environmental science has contributed the least is cognitive development and cognitive decline. Cognitive development in children was investigated using clinical assessment of cognitive functioning and motor development [53,81,114,116,120,121,122], or grade advancement [116]. A single study of the effects of noise on cognitive development used electrocardiography to assess infants’ autonomic reactions to noise as a measure of cognitive arousal [129]. Cognitive decline in elderly residents was monitored following a tsunami in Japan through questions aimed at testing memory, orientation, and pattern recognition [130].

#### 4.3.1. Evidence from Reviews (Climate Change, Flooding, Air Pollution, Urban Green Space)

Most reviews considered studies that were empirically interdisciplinary, which is consistent with the present scoping review. Papers included in the reviews also demonstrated the primary collection of environmental data, particularly pollution data within urban green spaces.

The measurement of mental health outcomes in climate change, flooding, air pollution, and urban green space varied depending on the type of the disorder. While depression and anxiety were commonly measured with self-report scales, substance abuse and suicide were commonly measured with hospitalisation records. It was suggested that the measurement of mental health needed to include more positive outcomes, for example post-traumatic growth [8]. Unlike our own review, many reviews examined the effects of environmental states on both physical health and mental health [84,90,93,94].

### 4.4. How Does the Relationship between Environmental Science and Mental Health Research Relate to Existing Evidence Linking Mental Health and Wellbeing to Demographic, Social, Economic, and Genetic Determinants?

The majority of the studies across the themes in our scoping review included one or more demographic variables, such as gender, age, or education. The social determinants (e.g., social class, community support) of mental health were assessed across themes, although there was some variation in which aspects were included. Economic variables were typically limited to employment or income, and inclusion of genetic determinants of mental health was rare and considered only through family history (natural disasters: [131]; noise: [43,76,123]; pollution: [115,120]). Between themes, natural disasters and natural environments studies had the widest coverage of additional determinants, with the inclusion of social, economic, demographic, and genetic determinants across studies. Pollution, oil spills, and wildfires also include contributions from social, economic, and demographic determinants; however, the variety of variables was more limited. Meteorological conditions, including temperature, are most limited in their consideration of additional determinants, including only community resilience (general meteorological conditions) and neighbourhood characteristics (temperature), in addition to demographic determinants (Table 8).

Social support (or lack thereof) can be an important determinant of mental health [24]. Disaster research considered social support in terms of the impact of social networks on general mental health, neurotic or mood disorders, and suicide or self-harm [109,132,133,134,135,136,137,138]. Social support was also considered for noise [44] and oil spills [133]. Social cohesion and contact [139,140,141] and group activities [142] were considered in studies of natural environments, often in relation to social spaces provided by natural environments. Social support may also be relevant at a more personal level and include partner violence, as considered in relation to natural disasters [108] and oil spills [55], or parental characteristics, as considered in relation to pollution [53,122]. Professional support was also considered in one study of wildfire impacts [138].

Community-level variables can also be considered social determinants of mental health, such as the presence of community support groups or organisations promoting community cohesion or a sense of belonging [24]. Community-level variables were included in a limited range of studies in this scoping review. Natural disasters typically have an impact at the community scale, and these studies have included community resilience [143], community attachment [143], perceived control, and optimism [132]. Noise research, which often involves the measuring or modelling of noise within the community, also incorporates neighbourhood [42,45,110,111,112] or community [76,78,144] characteristics. Village characteristics [116] and community variables [51] were also included. Strategies for coping with environmental change can also occur at a community level and impact individual mental health [56]. Studies focused on meteorological conditions rarely incorporated social determinants of mental health, with only a single paper considering community resilience [61].

Economic (or socio-economic) status can have an important influence on mental health, including income and employment status [145,146], as well as the ability to manage income. Income was widely incorporated into studies, with some studies also including more detailed measures such as changes in income [147,148,149], employment [149], or the ability to manage income [108,132]. Socio-economic (dis)advantage was also considered [77,120,141,150,151,152,153].

#### 4.4.1. Evidence from Review Studies (Climate Change, Flooding, Air Pollution, Urban Green Space)

There was limited information about existing determinants of mental health from previously published reviews. Review studies that were carried out at a global scale typically aimed to generate findings for policymakers and provided less detailed information. Those studies that were carried out at the local scale were more likely to discuss the existing socio-economic determinants of mental health, including young age [98], old age [87], social support [89], and minority status [88]. Children and the elderly were particularly vulnerable to air pollution that could adversely affect cognitive functions, and members of minority groups and people who lacked social support had a higher risk of developing mental illness after natural disasters caused by climate change.

Regardless of scale, most review studies—across climate change, flooding, air pollution, and urban green space themes—agreed on the importance of future research continually monitoring the mental health conditions of vulnerable populations, including older adults, children, and low-income workers [84,87,100]. The need to investigate mediating factors was also acknowledged, especially in air pollution research [10,98].

## 5. Discussion

This scoping review and synthesis of previously published review papers identified a large body of literature linking environmental science to mental health research; however, across the majority of studies, the direct contribution of environmental science was limited. Two key areas of mental health research would particularly benefit from the integration of environmental science: (i) the contribution of environmental factors to the origins and progression of mental health diseases and disorders; and (ii) the role the environment plays in the treatment of mental health and the promotion of good public health and mental wellbeing. A focus on both the positive and negative influences of the environment on mental health would also be valuable.

In this section, we discuss the limitations of the current research in relation to each research question. We develop a conceptual model to assist in addressing the final research question, identifying gaps and opportunities for future research. We integrate insight from our synthesis of reviews on climate change, flooding, air pollution, and urban green space as relevant.

### 5.1. What Is the Current Contribution of Environmental Science to Mental Health Research?

Across the themes and sub-themes identified within the scoping review, the contribution of environmental science to mental health research was limited, with studies often only considering the presence or absence of an event/environmental state or otherwise utilising crude measures of environmental exposure. Other previously published reviews have found the same, calling for the precise pathways involved to be better understood, as well as their relative importance across different timeframes [10]. Those environmental states related to chronic exposures covered in our review (i.e., noise, drought, chemical pollutant, oil spill) do provide some exceptions, with studies employing environmental monitoring and modelling of the environment at different spatial scales to consider the range of impacts (for example, see [76,77]).

Considering the studies reviewed and including those topics that have been the subject of recent reviews, the greatest opportunity for environmental science input is to develop processes at the beginning of the impact pathway. While this includes the measurement of environmental states and exposures that lead to mental health effects, environmental science can also provide insight on the upstream drivers and pressures that lead to changes in environmental states. Greater involvement of environmental science thus offers the potential to embed a wider systems perspective within mental health research, considering the drivers and pressures which lead to environmental change and shape relevant policy, going beyond the narrow focus on exposure-effect relationships. Additionally, greater integration of environmental science offers the opportunity to adopt more nuanced measures of exposure to improve understanding of exposure-effect relationships. We return to the issue of measurement in our discussion of methodological approaches below. There is also considerable opportunity for environmental scientists to bring new insights through the application of existing conceptual models of socio-ecological systems, such as the Intergovernmental Panel on Biodiversity and Ecosystem Services (IPBES) conceptual framework [154,155] to environment-mental health research. There is a need to move from the empirical interdisciplinarity demonstrated in the studies found in the scoping review to methodological and theoretical interdisciplinarity [39] at this interface to help inform interventions and solutions in terms of what works to address and mitigate the negative effects identified, including a focus on how, in what contexts, and for whom.

**Figure 4:**
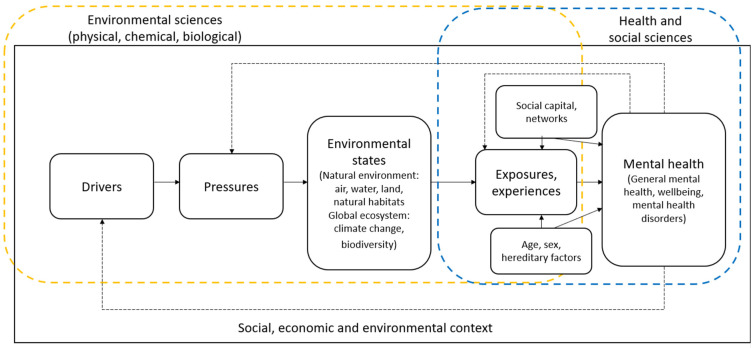
Conceptual model of environmental science and mental health nexus, drawing on the DPSEEA (Drivers, Pressures, State, Exposure, Effect, and Actions) framework ([27]; modified by [28,29] to include experiences) and Barton and Grant’s [25] Health Map for the local human habitat. This can be applied at the local to the global scale. Dotted lines indicate possible feedback loops, which should be considered in future work.

In light of the scoping review’s findings, we propose a conceptual model (Figure 4) to help structure the future interdisciplinary research agenda for environmental science and mental health. The model highlights the overlapping scope of environmental science and the health and social sciences within an interdisciplinary socio-ecological systems approach to researching mental health. It highlights the opportunity to consider the upstream determinants of mental health more widely through the direct involvement of environmental scientists in research collaborations.

#### 5.1.1. Future Research Focus: Expand Geographical Scope

The contribution of environmental science to mental health research within English-language publications in the past decade has largely occurred in North America, Europe, and Australia, and, with the exception of China, there have been few studies conducted elsewhere. Those themes in previously published reviews also identify geographic scope as a limitation, including noting China as a site for further research in the case of climate change [84]. Given the scale of ongoing environmental degradation and change globally and differences in environmental regulations, relationships to the environment, effects of environmental change, and cultural impacts (see, for example, reviews by [88,93], increasing research funding and capacity outside of these locations provides a valuable opportunity to increase scientific understanding and develop context-relevant and appropriate policy and innovation. Future reviews covering a wider range of languages, as well as greater efforts to address barriers to publishing in high-impact English-language journals (including publishing costs), would also be beneficial.

#### 5.1.2. Future Research Focus: Increase Range of Environmental Science Areas

The results show that whilst there is a broad coverage of themes at the nexus between the environment, environmental science, and mental health, research intensity is varied, with natural disasters having received more focused study compared with other themes identified in this scoping review. While there are myriad reviews of research on climate change (e.g., but not limited to [7,8,83,87,89]), partly due to calls by the Intergovernmental Panel on Climate Change stating that the link between climate change and mental health has been an understudied topic of great importance [97], few of these provide guidance on how to increase the contribution of environmental science, e.g., from presence/absence to data-rich research. Within the natural disasters theme, studies on wildfires were prevalent, whereas there were few studies addressing the mental health impacts of earthquakes and landslides. Similarly, within the pollution theme, oil spills were a common focus. The need to study the interconnected nature of environmental factors was also identified in previous reviews because environmental states are interconnected and do not exist in isolation [85,105].

### 5.2. What Are the Current Research Designs and Methodological Approaches Being Used in Environmental Science-Mental Health Research?

Designs were largely observational and often used secondary data, although in some themes (e.g., noise) field studies were also common. The analysis was predominantly quantitative and most often involved regression analysis, though there were several qualitative studies. This mirrored previously published reviews on flooding [93] and air pollution [10].

#### 5.2.1. Future Research Focus: Greater Application of Experimental Research Design Principles

A dominance of observational studies is perhaps not surprising, given that many of the studies in which environmental science contributes to mental health research occurred around natural disasters, oil spills, water contaminants, or meteorological conditions, which are impossible and/or undesirable to manufacture, and focus on negative impacts on mental health, which would be unethical to induce. Nevertheless, greater application of the principles of experimental design (as opposed to correlational analysis) through natural and quasi-experimental designs is necessary to improve the evidence base for causal effects on mental health. Secondary data sources, such as large-scale social surveys and administrative data, can prove highly valuable “before” data. There is also a significant opportunity for more widespread inclusion of control/comparator groups not exposed to environmental factors of interest, as many studies lacked appropriate counterfactual evidence. One area where there is greater opportunity for the application of randomised controlled experimental designs is in relation to the potential salutogenic effects of nature-based interventions such as forest schools, particularly in relation to identifying the attributes of environments that drive any observed mental health changes.

#### 5.2.2. Future Research Focus: Draw on Environmental Science to Include Better Measures of Exposure

Although natural disasters as a theme was most strongly represented in the scoping review literature, this body of research was noted for having minimal environmental science involvement. Measures of exposure were often limited (e.g., exposed vs. not exposed), a result also reported in recent studies on climate change and mental health [7]. A similar observation could be made for literature investigating pollution events such as oil spills or water contaminants. A greater involvement of environmental scientists in developing/selecting and applying appropriate measures or indices of exposure would permit a deeper understanding of “dose-response” relationships to be developed. “Dose-response” relationships were first described to explain the way in which drugs interact with the body to produce their effect at varied doses, with the understanding that this is often non-linear [156]. Although designed originally to aid in the correct dosage of prescription drugs, the same concept can be applied to environmental exposures and impacts on mental health, that is, at what level of exposure do either positive or negative effects begin to be seen, and how does this change as exposures are increased [157]. Such research would improve our understanding of mental health responses to environmental change and have practical implications for disaster recovery or the design of natural environments to promote good mental health.

Although published after the review described here and thus not included in our analysis, Merdjanoff and colleagues [158] provide one such suggestion for incorporating exposures into the study of natural disasters and mental health. Studying the impacts of Hurricane Sandy, their paper proposes a “Disaster Exposure Matrix”, which conceptualises exposures as either individual or community-level, direct or indirect. Through understanding how individuals are exposed, the paper finds an increase in the likelihood of developing PTSD with increasing levels of direct individual exposure but not for any level of indirect individual or direct or indirect community exposure [158]. Examples of such efforts could also be drawn from studies investigating the effect of air pollution on mental health (for a review, see [10]). There is also considerable potential for interdisciplinary conversations drawing together environmental scientists and mental health researchers to explore the constructs of *exposure* and *experience* (Figure 4) in their relation to mental health. This might be particularly relevant in addressing the highlighted issues of exposure measurement around natural disasters and extreme weather events, where experiences of trauma and loss play an important mediating role in mental health outcomes.

#### 5.2.3. Future Research Focus: Inclusion and Development of Mixed and Qualitative Methods

The mixed methods paradigm offers a structured route through which to integrate qualitative and quantitative approaches to generate a richer understanding of a research area. Qualitative approaches help build depth and breadth of understanding, provide holistic insight into an individual’s experience, and identify possible research directions for quantitative research (e.g., [124]). These approaches can help give a holistic view of the experiences of individuals and communities and the culturally specific aspects of mental health and wellbeing. A mixed-methods approach could help address the challenges of bringing different research traditions together. Such integration would call for interdisciplinary project teams that incorporate the social, environmental, and health sciences from the project’s conception and the building of a shared understanding amongst team members of the value of different types of evidence and research methods in contributing to knowledge on the environment and mental health. On a wider scale, funders, career progression, and journal scope would also need to continue to be adapted to promote and reward such interdisciplinary work. This type of research, alongside researchers in environmental science and mental health domains, would allow “the key role social science can play in a holistic and critical analysis of environment and health interactions” [159] (p. 1) to be incorporated into studies of social-ecological interactions between the environment, environmental science, and mental health outcomes. This would enable studies to move from empirical interdisciplinarity, which currently dominates, to theoretical interdisciplinarity [39].

#### 5.2.4. Future Research Focus: Longitudinal Analysis

Most studies identified through the scoping review took a cross-sectional approach to investigating the relationship between environment and mental health. Other previously published reviews also highlighted the predominance of cross-sectional designs in this research area, confirming the need for more longitudinal analysis [7,60,93,102,105]. Longitudinal studies: (i) show the impact and consequences [8] of a change in environmental states over time and before an exposure; (ii) allow a more nuanced understanding of environmental exposure throughout the life course; (iii) recognise how different disorders and vulnerabilities can manifest at different life stages; (iv) help understand causal relationships and understand mental health outcomes; and (v) facilitate examination of the complexities of multiple different types of environmental exposures and how they interact [85]. Natural experiments offer opportunities for longitudinal studies to integrate environmental science and mental health. The characteristics of many of the environment-related issues considered within the identified literature (e.g., natural disasters, oil spills) mean that understanding of pre-event mental health is limited. Investment in supporting interaction between environmental scientists and mental health researchers in the development of longitudinal datasets—including through improved data linkage supported by the latest environmental science methods—may provide the opportunity to understand the impacts of such events more fully.

### 5.3. How Does the Relationship between Environmental Science and Mental Health Research Relate to Existing Evidence Linking Mental Health and Wellbeing to Demographic, Social, Economic, and Genetic Determinants?

In general, studies took little account of additional determinants of mental health or the influence of wider socio-economic and political systems. The limited incorporation of the wider context of mental health can silo the contribution of environmental science to mental health research and restrict the applicability of findings, especially for policymakers.

#### 5.3.1. Future Research Focus: Integration of Multiple Conceptual Models

This scoping review highlighted that, overall, the range of potential confounding variables included in models was somewhat limited (see also [7,10]). Related to this, there was also limited evidence of environmental exposure perspectives being integrated with other conceptual models of mental health (e.g., genetic or social determinants of mental health). This highlights the scope to use conceptual models that include both environmental science and mental health theory explicitly to develop and improve our understanding of the relationships between environmental science and mental health, the causal pathways involved, and to what extent these pathways interconnect [10]. For example, in the theme of natural disasters and wildfires, greater incorporation of coping mechanisms and other factors underpinning resilience, as well as a strengthening of the evidence base around which interventions can help limit mental health impacts, would be valuable (also found by [8]). Understanding would additionally be furthered by considering the physiological pathways that link environmental exposures, in their broadest sense, to mental health. The creation of conceptual models that bridge the disciplinary gap would enable an informed consideration of the potential options for mitigation based on the physiological adaptations achievable through mitigation of the environmental stressor and the time and spatial scales over which impacts and adaptations occur.

#### 5.3.2. Future Research Focus: Consideration of Socio-Economic (Political) Systems

The environmental and socio-economic (political) determinants of mental illness are interrelated. For example, poorer neighbourhoods, whose inhabitants may have job insecurity or poor working conditions, are also often subjected to poor quality environments (e.g., [124]), thereby increasing health injustice burdens. There may also be potential for reverse causality, whereby those with worse mental health are marginalised to areas more likely to have poorer resilience to climate change and poorer environmental states [7]. In addition, mounting evidence suggests that neoliberal free market policies leading to, amongst other things, income inequality, worker disempowerment, and inadequate social systems, may be fueling increased levels of poor mental health in the United States [160]. Correlation studies demonstrate that unequal rich countries have higher prevalence of poor mental health than more equal rich countries [161]. These same economic systems and policies, prevalent in the global north and heavily reliant on high levels of consumption and production, appear to not only negatively impact their own nation’s mental health but also have wider environmental and social impacts (e.g., spillovers from tele-coupling effects) on other nations, usually poorer ones in the global south [162]. This multitude of wide-reaching environmental and social impacts, such as the loss of land rights and associated livelihoods, increased flooding, wildfires, environmental pollution, and sea-level rises, all exacerbate already existing social and environmental global injustices and inequalities, negatively affecting human health and wellbeing. This necessitates considering the different spatial impacts on mental health and wellbeing within the nexus of environmental science and mental health research. It would also be prudent to investigate and integrate spatial impacts with global systemic frameworks, for example, IPBES [162] and the “doughnut of social and planetary boundaries” [163], to better incorporate societal values and thresholds, including wellbeing, into environmental and planetary boundaries.

### 5.4. What Are the Evidence Gaps and Opportunities for the Contribution of Environmental Science to Mental Health Research?

There have been several recent reviews looking at specific aspects of environmental science and mental health research. We have taken this work further by integrating the findings of these theme-level reviews with an in-depth scoping review considering the wide range of environmental science contributions to mental health, and we detail here the recommendations for our final research question, identification of the gaps and opportunities for future research.

#### 5.4.1. Future Research Focus: Considering ‘Good’ Mental Health

The scoping review found limited attention paid to the ways in which environments can support maintenance of “good” mental health or promote wellbeing as “more than just the absence of mental disorders” [164]. There has been recent interest in maintaining good mental wellbeing independent of or to prevent development of mental health disorders [8,165,166]. The COVID-19 pandemic has highlighted the importance of maintaining good mental health to provide resilience to personal or collective distressing events [167] and the potential role of natural environments (e.g., [168]). Environmental science would provide a valuable contribution in identifying environmental situations where good mental health can be promoted and how the impacts of negative environmental states may be mitigated. More work is required that focuses on specific policy objectives and interventions that could help policymakers and practitioners (e.g., planners) operationalise findings [105]. Such research can contribute to initiatives such as green prescribing. Research is also needed that quantifies the costs and mental health benefits of these types of public health strategies [93].

#### 5.4.2. Future Research Focus: Exploring Variation between and within Communities

Different population groups include a range of socio-economic status, demographics, and pre-existing illnesses [8,93]. This review and previous review papers have noted the likely different mental health impacts of environmental factors on indigenous populations, displaced groups, and other marginalised populations with strong links to the land [8,83,88,89,93], children [92], older people [98], and workers [96]. At the study level, the often local but coarse scale of research (e.g., a single community with only minimal consideration of variation in environments or exposure within the community) limits understanding of the impacts of exposures on different population groups (e.g., gender-disaggregated analyses) and how socio-demographic factors might moderate the impact of exposures. This limitation has also been recognised in a review of studies of climate change and mental health [7]. Comparative, place-based analyses would help address this gap, provide contextual understanding of results, and therefore improve the potential to transfer results to different locations.

#### 5.4.3. Future Research Focus: Review of Mediating Pathways

Although we have considered the broad range of environmental science, our review has not been able to create a holistic overview of the mediating pathways by which various environmental risks and protective factors might influence mental health. This has been called for widely across the literature (e.g., [14,169], as well as [105]). While this would be a significant undertaking, necessitating interdisciplinary working and substantial resourcing, we anticipate that it would enable substantive advances in the contributions of environmental science to mental health research. Such research would additionally promote a greater holistic understanding of health and wellbeing as proposed in Barton and Grant’s [25] Health Map, with the potential of integrating with global sustainability “good life” conceptual models such as those developed by IPBES [155,162].

## 6. Limitations

Although we searched both general (Web of Science) and health-specific (PubMed) databases of the published literature and a range of grey literature sources, our review may not have identified all mental health research that included environmental science, particularly those within specialist databases (e.g., PsycInfo) or grey literature outside of the EU. Due to the volume of literature and our desire to focus on the most up-to-date sources of information, we limited our review to studies published after 2010 up to 2020. Earlier work linking environmental science to mental health has therefore been excluded, though it may provide insight into how the disciplines might interact and should be considered where specific environmental science and mental health linkages are being researched. We similarly appreciate that there may be relevant insight in research published after our census period. Given, however, the influence of the COVID-19 pandemic on the discourse around links between the environment and mental health, we believe it is important to only include literature prior to the start of the pandemic. Our review makes an important contribution by assessing the evidence base prior to this unprecedented natural experiment in the environment and mental health research arena. Further, we used only broad mental health terms and did not focus on specific disorders. While this enabled us to map the breadth of environmental science contributions to mental health research, we are not able to explore the details of specific disorders.

We also limited our review by excluding primary papers on environmental science topics that had been the subject of recent reviews linked to mental health. Although summaries of those review topics have been included throughout, they may contain exemplar studies that have not been identified and may provide valuable insight into how environmental science can contribute to mental health research. An additional limitation in relation to summarising reviews is that these did not always have the depth of information we were able to extract from the empirical studies that formed part of the main scoping review.

## 7. Conclusions

The intersection of environmental science and mental health research is clearly fundamental, as evidenced by the over 200 papers included in this scoping review. Most of the papers, however, had a stronger mental health focus than an environmental science focus. One of the original contributions of this paper is the development of a conceptual model, which provides a framework for the more substantive involvement of environmental science to strengthen measurement (e.g., moving towards dose-response relationships and beyond simple presence/absence of an environmental state) and facilitate a deeper understanding of potential causal relationships. Over the past decade, there has been a greater focus on poor mental health than on maintaining or improving good mental health and wellbeing, with most studies limited to a single point in time. This continued emphasis on the environmental risks and hazards for mental health is relevant for addressing global challenges, yet this focus leaves out critical insights around the benefits of our everyday relationships with our surrounding environments. These insights are needed to generate and evaluate environmentally focused solutions. This review has demonstrated that environmental science indeed makes varied contributions to mental health research. We suggest that further gains would be made through the development of a community of practice between researchers of these specific disciplines, which in turn could benefit mental health across populations.

## Figures and Tables

**Table 1:** Search strings used in the Web of Science and PubMed.

Mental Health Keywords.		Environmental Science Keywords		Exclusions
“mental health” OR “mental ill*” OR “mental disorder” OR “mental health and wellbeing” OR “mental wellbeing” OR “cognitive development” OR “cognitive decline” OR “psychopatholog*”	AND	flood* OR drought OR wildfire OR “bush fire” OR “forest fire” OR landslide* OR “climate change” OR “global warming” OR landscape OR noise OR soundscape OR pollut* OR biodivers* OR tree* OR forest* OR wood* OR wild* OR “natural environment” OR “natural land” OR “natural space” OR “natural area” OR “air quality” OR “water quality” OR ecosystem OR lake OR river OR coastal OR erosion	NOT	mice OR mouse OR rat* OR rodent* OR drosophila

* indicates truncation (wild-card) operator used (e.g., pollut* will identify all terms beginning with the sequence “pollut”, including pollution, polluting etc.).

**Table 2:** Data charted by research question.

Research Question	Data Extracted
What is the current contribution of environmental science to mental health research?	Environmental science topic
	Mental health area
	Interaction between environmental science and mental health ^1^
	Measures (e.g., mean temperature, presence of oil spill, and prescription rates)
	Geographic location of study
	Study population characteristics (e.g., population, study sample size)
What are the current research designs and methodological approaches being used in environmental science-mental health research?	Methods (e.g., rainfall records, self-report questionnaire)
	Study design (lab, field, and secondary data)
	Analysis (e.g., regression, ANOVA, and content analysis)
How does the relationship between environmental science and mental health research relate to existing evidence linking mental health and wellbeing to demographic, social, economic, and genetic determinants?	Additional determinants/variables
What are the evidence gaps and opportunities for the contribution of environmental science to mental health research?	Future research suggested by study authors

^1^. Categories for defining interactions between disciplines are based on [39].

**Table 3:** Themes within which environmental science contributes to mental health research.

Theme (Sub-Theme)	Barton and Grant [25] Domains	Description	No. of Papers	
**From scoping review**				
Natural disasters (general)	Air, climate change, land, and water	A catastrophic natural event (e.g., hurricane, drought, landslide), excluding fire.	50	
Natural disasters (wildfire)	Climate change and land	A specific type of natural disaster caused by fire.	20	
Noise	n/a	Natural or anthropogenic noise.	36	
Pollution (general)	Air, land, and water	Chemical additions to environment, excluding oil spills.	19	
Pollution (oil spill)	Water	Chemical addition to environment in form of oil spill.	14	
Natural environments	Biodiversity, land, natural habitats, and water	Environments containing natural features, although may have varied levels of anthropogenic influences (e.g., agricultural land).	32	
Meteorological conditions (general)	Air, climate change, and water	Typical weather conditions experienced seasonally, monthly to daily such as fluctuations in humidity and rainfall, except temperature. More intensive storm and rainfall events (e.g., Typhoons, hurricanes) were covered in Natural disasters ^1^ (general).	7	
Meteorological conditions (temperature)	Air and climate change	Typical temperature conditions experienced seasonally, monthly to daily.	16	
**From reviews**				
Climate Change	Climate change	Change in global or regional climate conditions	n/a ^2^	
Flooding	Water and climate change	Inundation of normally dry land by large volumes of water	n/a ^2^	
Air pollution	Air	Chemical additions to the atmosphere	n/a ^2^	
Urban green space	Land, natural habitats, and biodiversity	Natural environments in urban areas, predominately publicly accessible spaces	n/a ^2^	

Note. Papers may appear in more than one category if they study multiple themes (e.g., the impacts of pollution and noise on general mental health). ^1^. We recognise that “natural disaster” may overshadow the human element associated with these events [41] however, we have chosen to use the term “natural disaster” throughout as we believe it to be widely recognised across disciplines. ^2^. n/a = Not applicable—these themes were the subject of previously published review articles and so were not included in the main scoping review (review papers are reported in Section 4.2.1, Section 4.3.1 and Section 4.4.1).

**Table 4:** Mental health research areas covered in our scoping review. Mental health disorders were categorised and defined in accordance with the International Classification of Diseases and Related Health Problems, 10th revision (ICD-10; [40]) and cognitive development and decline [82].

Mental Health Area	ICD-10 Classification	Definition	No. of Papers
General mental health	NA	Day-to-day mental health, not linked to specific disorder.	100
Wellbeing	NA	Conceptualised as a subjectively experienced positive mental state (see Box 1).	27
Schizophrenia and delusional disorders	F20–F29	Including chronic, acute, and transient psychotic disorders, of which schizophrenia is the most prominent disorder.	3
Mood disorders	F30–F39	Disorders in which the fundamental disturbance is a change in affect or mood to depression (with or without associated anxiety) or to elation.	56
Neurotic disorders	F40–F48	Include anxiety, stress, obsessive-compulsive disorder, and dissociative disorders.	30
Behavioural disorders	F50–F69 and F90–F98	Conditions and behaviour patterns of clinical significance which tend to be persistent and appear to be the expression of the individual’s characteristic lifestyle and mode of relating to themselves and others. Including sleep, eating and sexual disorders.	4
Substance abuse	F10–F19	Disorders attributable to the use of one or more psychoactive substances.	8
Disorders of adult personality and behaviour	F60–F69	Severe disturbances in the personality and behavioural tendencies of the individual.	0
Mental retardation	F70–F79	A condition of arrested or incomplete development of the mind.	0
Disorders of psychological development	F80–F89	Disorder with onset during infancy or childhood involving impairment or delay in development of functions that are strongly related to biological maturation of the central nervous system.	0
Suicide or self-harm	X60–X84	Purposefully self-inflicted poisoning or injury.	7
Cognitive development	NA	Development of knowledge acquisition and application. Including memory, problem solving, reasoning, and executive function.	7
Cognitive decline	NA	Decline of knowledge acquisition and application. Including memory, problem solving, reasoning, and executive function.	2

Note. Papers may appear in more than one category if they studied multiple themes (e.g., the impacts of pollution on general mental health and depression). Note that papers reporting studies on climate change, flooding, air pollution, and urban green space were assessed via charting and extraction of review articles considering these topics and are not included here (review papers are reported in Section 4.2.1, Section 4.3.1 and Section 4.4.1).

**Table 5:** Number of papers identified through scoping review by environmental science theme and mental health area.

Theme (Sub-Theme)	General Mental Health	Mental Wellbeing	Schizophrenia and Delusional Disorders	Mood Disorders	Neurotic Disorders	Behavioural Disorders	Substance Abuse	Suicide or Self-Harm	Cognitive Development	Cognitive Decline
Natural disasters (general)	28	0	0	16	19	4	3	4	0	1
Natural disasters (wildfire)	9	3	1	11	15	1	3	2	0	0
Noise	19	1	0	12	9	3	0	3	1	0
Pollution (general)	5	1	1	5	3	1	1	2	7	0
Pollution (oil spill)	5	3	0	7	8	0	0	2	0	0
Natural environments	15	7	0	10	10	1	0	0	0	0
Meteorological conditions (general)	5	0	2	2	1	0	0	1	0	0
Meteorological conditions (temperature)	12	1	1	3	3	0	1	1	0	1

Note. Categories are not exclusive, and one paper may cover more than one environmental science theme or mental health area. Review papers are reported in Section 4.2.1, Section 4.3.1 and Section 4.4.1.

**Table 6:** Scope of research covered by review papers on climate change, flooding, air pollution, and urban green space.

Review Topic	Paper	Years Covered	Including Grey Literature	Geographic Scope	Mental Health Areas
Climate change	Berry et al., 2011 [83]	Not specified	Yes	Global	Depression, anxiety, psychosis, Post-Traumatic Stress Disorder (PTSD), and suicide
	Chan et al., 2019 [84]	2000–2018	Yes	China	General mental health and PTSD
	van den Bosch and Meyer-Lindenberg, 2019 [85]	Not specified	Yes	Global	Depression and suicide
	Veenema et al., 2017 [9]	Not specified	No	Global	General mental health
	Hayes et al., 2019 [86]	2000–2017	Yes	Global	Substance abuse, depression, anxiety, PTSD, and suicide
	Hayes and Poland, 2018 [8]	2000–2017	Yes	Global	substance abuse, depression, anxiety, PTSD, and suicide
	Kinay et al., 2019 [87]	2000 onwards	Yes	China	General mental health
	Jaakkola et al., 2018 [88]	1990–2017	Yes	Canada	Mental wellbeing
	Patz et al., 2014 [89]	2009–2014	Yes	Global	General mental health
	Dannenberg et al., 2018 [90]	Not specified	Yes	Global	General mental health
	Yusa et al., 2015 [91]	1993–2013	Yes	Global	Depression and suicide
Flooding	Berry et al., 2011 [83]	Not specified	Yes	Global	Depression, anxiety, psychosis, PTSD, and suicide
	Chan et al., 2019 [84]	2000–2018	Yes	China	General mental health and PTSD
	Veenema et al., 2017 [9]	Not specified	No	Global	General mental health
	Garcia and Sheehan, 2016 [92]	to 2015	Yes	Global	General mental health
	Burton et al., 2016 [93]	2005–2015	Yes	Canada	General mental health and PTSD
	Du et al., 2010 [94]	1998 onwards	Yes	Global	General mental health
	Mousavi et al., 2020 [95]	to 2017	Yes	Iran	General mental health
	Schulte et al., 2016 [96]	2008–2014	Yes	Global	General mental health
	Verner et al., 2016 [97]	1990–2014	No	Global	General mental health
	Stanke et al., 2012 [98]	2004–2010	No	Global	General mental health
Air pollution	van den Bosch and Meyer-Lindenberg, 2019 [85]	Not specified	Yes	Global	Depression and suicide
	Braithwaite et al., 2019 [10]	1974–2017	No	Global	Psychiatric disorder, depression, anxiety, bipolar disorder, psychosis, and suicide
	Tzivian et al., 2015 [99]	Not specified	Yes	Global	Anxiety, mood disorders, cognition, Alzheimer’s, and cognitive decline
	Bos et al., 2014 [100]	2009–2013	No	Global	Cognition
	Cipriani et al., 2018 [101]	to 2017	Yes	Global	Cognition, cognitive decline, Alzheimer’s, and dementia
	de Prado Bert et al., 2018 [102]	to 2017	Yes	Global	Cognitive development
	Buoli et al., 2018 [103]	1982- 2018	Yes	Global	General mental health schizophrenia and delusional disorders, depression, anxiety, Attention Deficit Hyperactive Disorder (ADHD), autism, and suicide
Green space	van den Bosch and Meyer-Lindenberg, 2019 [85]	Not specified	Yes	Global	Suicide and depression
	Gladkikh et al., 2019 [104]	to 2018	Yes	Global	General mental health
	Hankey and Marshall, 2017 [105]	Not specified	Yes	Global	Depression, anxiety, and cognitive decline
	Kabisch, 2019 [12]	2013 onwards	No	Global	General mental health and wellbeing

**Table 7:** Research approaches and analysis across environmental science themes and the mental health area. Colours indicate mental health area groupings; dark grey squares indicate where designs and analysis have been used. Note that because they have been subject to recent reviews, climate change, flooding, air pollution, and urban green space are not considered here but have been described in the text.

		Quantitative													Qualitative						Quantitative													Qualitative				
Cognitive and Mental Health Area		Descriptive	Correlation	ANOVA	Regression	Delphi	Path Modelling/SEM	Bayesian Modelling	Case Crossover	Principal Component Analysis	Latent Class	Mediation Modelling	Risk Ratio	Chi Squared	Content Analysis	Phenomenological	Thematic Analysis	Grounded Theory	Mixed		Descriptive	Correlation	ANOVA	Regression	Delphi	Path Modelling/SEM	Bayesian Modelling	Case Crossover	Principal Component Analysis	Latent Class	Mediation Modelling	Risk Ratio	Chi Squared	Content Analysis	Phenomenological	Thematic Analysis	Grounded Theory	Mixed
		**Natural disasters** (general)																			**Natural disasters** (wildfire)																	
General mental health																																						
Wellbeing																																						
Substance abuse																																						
Schizophrenia and delusional disorders																																						
Mood disorders																																						
Neurotic disorders																																						
Behavioural disorders																																						
Suicide or self-harm																																						
Cognitive development																																						
Cognitive decline																																						
		**Pollution** (general)																			**Pollution** (oil spill)																	
General mental health																																						
Wellbeing																																						
Substance abuse																																						
Schizophrenia and delusional disorders																																						
Mood disorders																																						
Neurotic disorders																																						
Behavioural disorders																																						
Suicide or self-harm																																						
Cognitive development																																						
Cognitive decline																																						
		**Noise**																			**Natural environments**																	
General mental health																																						
Wellbeing																																						
Substance abuse																																						
Schizophrenia and delusional disorders																																						
Mood disorders																																						
Neurotic disorders																																						
Behavioural disorders																																						
Suicide or self-harm																																						
Cognitive development																																						
Cognitive decline																																						
		**Meteorological conditions** (general)																			**Meteorological conditions** (temperature)																	
General mental health																																						
Wellbeing																																						
Substance abuse																																						
Schizophrenia and delusional disorders																																						
Mood disorders																																						
Neurotic disorders																																						
Behavioural disorders																																						
Suicide or self-harm																																						
Cognitive development																																						
Cognitive decline																																						

**Table 8:** Additional determinants are considered in studies by environmental science theme and mental health area. Colours indicate mental health area groupings; dark grey squares indicate where determinants have been used. Note that because they have been subject to recent reviews, climate change, flooding, air pollution, and urban green space are not considered here but have been described in the text.

		Social					Economic				Demographic						Genetic		Social					Economic				Demographic						Genetic
Cognitive and Mental Health Domains		Social Class	Neighbourhood Characteristics	Community Resilience	Relationship Status	Social Contact and Support	Access to Car	Working Status	Quality of Life	Income	Gender	Age	Education	Health and Lifestyle	Children	Ethnicity	Family History		Social Class	Neighbourhood Characteristics	Community Resilience	Relationship Status	Social Contact and Support	Access to Car	Working Status	Quality of Life	Income	Gender	Age	Education	Health and Lifestyle	Children	Ethnicity	Family History
		**Natural disasters** (general)																	**Natural disasters** (wildfire)															
General mental health																																		
Wellbeing																																		
Substance abuse																																		
Schizophrenia and delusional disorders																																		
Mood disorders																																		
Neurotic disorders																																		
Behavioural disorders																																		
Suicide or self-harm																																		
Cognitive Development																																		
Cognitive Decline																																		
		**Pollution** (general)																	**Pollution** (oil spill)															
General mental health																																		
Wellbeing																																		
Substance abuse																																		
Schizophrenia and delusional disorders																																		
Mood disorders																																		
Neurotic disorders																																		
Behavioural syndromes																																		
Suicide and self-harm																																		
Cognitive Development																																		
Cognitive Decline																																		
		**Noise**																	**Natural environments**															
General mental health																																		
Wellbeing																																		
Substance abuse																																		
Schizophrenia and delusional disorders																																		
Mood disorders																																		
Neurotic disorders																																		
Behavioural syndromes																																		
Suicide and self-harm																																		
Cognitive Development																																		
Cognitive Decline																																		
		**Meteorological conditions** (general)																	**Meteorological conditions** (temperature)															
General mental health																																		
Wellbeing																																		
Substance abuse																																		
Schizophrenia and delusional disorders																																		
Mood disorders																																		
Neurotic disorders																																		
Behavioural syndromes																																		
Suicide and self-harm																																		
Cognitive Development																																		
Cognitive Decline																																		

## Data Availability

Data associated with this review are available in the Supplementary Materials.

## Supplementary Materials

The following supporting information can be downloaded at: https://www.mdpi.com/article/10.3390/ijerph20075278/s1.
