# Supplementary material for: The Contribution of Environmental Science to Mental Health Research: A Scoping Review
Source: Int J Environ Res Public Health. 2023 Mar 27;20(7):5278. doi: 10.3390/ijerph20075278 (PMC10094550; doi:10.3390/ijerph20075278)
Supplement: Supplementary file 1 [file ijerph-20-05278-s001.zip › Protocol - Supplementary Material.pdf]

Scoping review protocol

Michaela Roberts, Katherine N Irvine, Antonia Eastwood, Kathryn Colley, Mags Currie and Zulin Zhang

Background

Mental health is governed by complex, interacting factors: social, economic, demographic, genetic, experiential and environmental. There is therefore a general move to a more holistic approach to managing mental health, including cognitive development, healthy aging, disorders (e.g. anxiety, depression) and subjective experiences of mental wellbeing. A considerable body of research on mental health resides within the social and psychological disciplines including potential salutogenic effect of time spent in natural environments. This review specifically seeks to understand the role that environmental science (ecological/biological, physical, chemical) can play in mental health research, identifying where synergies exist, highlighting gaps towards which future research might be directed, and informing strategic planning within the Natural Environment Research Council (NERC).

Review Method rationale

A scoping review methodology takes a systematic approach to create an evidence synthesis to clarify concepts, and identify types of evidence, key research characteristics and evidence gaps to inform future research (Munn et al. 2018). Suited to open exploratory questions, scoping reviews can account for varied research designs as found in environment-mental health research. They are more systematic and extensive than a literature review, but do not critically appraise the evidence for intervention as in a full systematic review. Our approach follows Arksey et al 2005, Collins et al 2015 and Sucharew et al 2019 and will be reported according to PRISMA guidelines for scoping reviews (http://www.prisma-statement.org/Extensions/ScopingReviews).

Objective

The primary objective of this review is to understand the potential contribution that environmental science can make to mental health research and innovation, and to identify evidence gaps and opportunities. This is addressed through four questions:

- What is the current contribution of environmental science to mental health research?
    - This includes consideration of: the pathways by which the environment impacts on mental health; how environmental science has been leveraged to understand these pathways or impacts; how the communication of environmental science impacts on mental health
- How does the relationship between environmental science and mental health research relate to existing evidence linking mental health to social, economic, demographic and genetic determinants?
- What are the current research designs and methodological approaches being used in environmental science-mental health research?
- What are the evidence gaps and opportunities for the contributions of environmental science to mental health research?

Scope

	Scope	Rationale
Geographic area	Global	We anticipate that relevant research is being conducted worldwide. Although the review's main goal is to provide information for future NERC strategic planning, we may expect to find useful examples from elsewhere which may be applicable to the UK. NERC also has limited international and UK Overseas Territories calls.
Language	English	Limitations of the review team.
Time period	Post-1990 **Amendment to protocol:** The high volume of literature meant that the date range was further reduced to consider papers from 2010 onwards only.	A thirty-year time frame will enable progression of the topic to be followed, but will maintain a focus on current research. Limiting the time frame is needed to fit the resources available for the review. In designing future NERC strategic planning are most concerned with current research.
Definition of environmental science	Wide and covers physical, chemical and biological elements of environmental science.	Broad coverage of environmental science is needed to capture the range of potential areas where mental health and environmental science research may intersect.
Definition of mental health	Covering subjective wellbeing, cognitive development ageing, and psychiatric disorders.	Broad coverage of mental health as with environmental science to capture the range of areas where it may intersect with environmental science research. Stemming from WHO definition of health as: "state of complete physical, mental and social well-being and not merely the absence of disease or infirmity".

Methods

Keywords

	Central keywords	Potential keywords	Rationale
Mental health	Mental Health; Wellbeing; Cognitive Development; Cognitive Decline; Psychiatric Disorders;	Broad: Planetary health, psychosocial, solatalgia, ecological anxiety/grief, climate anxiety/grief<br><br>Relating to wellbeing: Quality of Life, life satisfaction, general health questionnaire, relaxation, self esteem, psychological restoration, stress, destress.<br><br>Relating to cognitive development: Autism, ADD, ADHD, neurocognitive disorder, behavioural problems, conduct disorder<br><br>Relating to cognitive decline: Dementia, Alzheimer's	Developed from WHO ICD list of mental and behavioural disorders, focused on those most commonly diagnosed disorders, and which are most often covered in reviews of mental

		Relating to psychiatric disorders: Anxiety, Depression, Mood Disorders (bipolar), general psychopathology score, revised clinical interview schedule, seasonal affective disorder, schizophrenia, schizotypal disorder, schizoaffective disorder, delusional disorder, psychotic disorders, psychotic symptoms, psychotic experience, trauma, externalising symptoms, internalising symptoms, phobia, panic disorder, OCD, PTSD, dissociative disorder, dysthymic disorder, mania, neurosis, suicide, self harm, substance abuse.	health, with additional keywords added by expert consultation. Also including wellbeing and development as defined in the commissioned call for a review.
Physical Environment	Flooding; Drought; Fire; Heat; Landslides; Particulate Matter	Natural Disaster; Soil quality, water stress, water level, water inundation, climate change, riparian corridor, water, river, coast, mountain, beach, dune, waterfalls, rock pools, stacks, arches, cliffs, geomorphology, geomorphic hazards, noise, avulsions, sedimentation, rain, sun, daylight, storm, hurricane, cyclone, erosion, sea level rise, aesthetics, landscape, weather, tsunami, earthquake, seasonality.	Developed from Barton and Grant 2006 conceptual model and consultation with experts through workshops.
Chemical Environment	Air pollution; Water pollution; Neurotoxicity;	Soil quality; Heavy metals; micro-plastics; endocrine disruptors, acidification, carbon monoxide, nitrogen oxides, sulphur dioxide, polycyclic aromatic hydrocarbons, aeroallergen, ozone	
Ecological/ Biological Environment	Biodiversity; Nature;	Invasive species; Disease; Pathogens; trees, wilderness, Normalised difference vegetation index, wildlife, forest, bluespace, greenspace, land use	

All searches in all databases are recorded into a spreadsheet, including rationale for either using the outcome of the search, or direction of further refinement.

Date	Where searched	Search string	Number of papers	Accepted?	Rationale	Comments
Date of search	Name of database	Exact search string used	Number of papers found with search string	Is this the final search string?	Why string accepted or rejected, including edits made to next string	Additional comments

Once a search has been accepted all papers, regardless of whether they are later screened out, are entered into paper database. Duplicates are identified and removed from final paper count.

Paper ID code	Authors	Date	Title	Publication	Link	Database	Duplicate?
Author name and year – paper					DOI to paper	Where extracted from	Is this a duplicate record from

saved as this in folder							another database?

Where searched

Database	Rationale	Link
Web of Science	Comprehensive interdisciplinary database	https://apps.webofknowledge.com
PubMed	In-depth medical database	https://pubmed.ncbi.nlm.nih.gov/
UK Government Research and Statistics	Grey literature of UK Government research	https://www.gov.uk/search/research-and-statistics
Scottish Government Publications	Grey literature of Scottish Government research	https://www.gov.scot/publications/
Welsh Government Research and Statistics	Grey literature of Welsh Government research	https://gov.wales/statistics-and-research
Northern Ireland Research and Statistics Agency	Grey literature of Northern Ireland Government research	https://www.nisra.gov.uk/
EU Public health publications	Grey literature of EU publications	https://ec.europa.eu/health/publications_en
British Library	Identify NGO reports from UK based organisations	http://explore.bl.uk/primo_library/libweb/action/search.do?vid=BLVU1

Inclusion and exclusion criteria

	Criteria	Rationale
Geography	No exclusions.	See scope. Should the volume of literature identified be beyond the limitations of available resources for the review, the geographic scope may be modified, e.g. to Northern Europe.
Climate	No exclusions.	The intersection between environmental science and mental health research is unlikely to be varied by climate, although outcomes of the relationship may be impacted by differences in climate.
Date	Exclude papers pre-1990 **Amendment to protocol:** The high volume of literature meant that the date range was further reduced to consider papers from 2010 onwards only.	See scope. Should the volume of literature identified be beyond the limitations of available resources for the review, the date range may be modified (e.g. to last decade).
Population	Human subjects only	Excluding studies of brain development or drug treatment on other model organisms. The mental health definition for this research includes childhood development and aging, therefore all ages are relevant to the question. The interaction between environmental science and mental health research would be expected to be relevant to both urban and rural populations, across all demographic, social and economic characteristics.

Methods	Incorporating both mental health and environmental science in the methods, excluding papers that only include links in discussions. Including both qualitative and quantitative methods.	The review is concerned with the state of interdisciplinarity between mental health and environmental science research, including positive and negative outcomes for mental health. Papers which only touch on the other discipline in the discussion, but do not incorporate into the methods, would not be considered interdisciplinary.
Type of outcome	Association between changes in environmental science and mental health.	As NERC funded this review is focused on impacts that environmental science research can have for mental health research, therefore outcomes should be measured in terms of mental health. Papers which only measure environmental science in a mental health context (e.g. measuring levels of pollutant thought be to connected to development) but do not measure change should be excluded, but the reason noted.
Type of paper	Exclude review, opinion and descriptive.	Reviews are excluded from the systematic review to avoid double counting of original papers, but will be used for background knowledge, and reference lists used to identify papers.<br><br>Opinion and descriptive papers are excluded because they do not represent a robust testing or exploration of mental health and environmental science research. However, they may form part of the background to the research.
Mental health	Following WHO definition of health as: "Health is a state of complete physical, mental and social well-being and not merely the absence of disease or infirmity". To include therefore wellbeing, cognitive development and decline as well as disorders.<br><br>Focused on most common disorders of anxiety, depression and mood disorders.<br><br>Considering only individual mental health, and considering sustained change, not momentary (e.g. I feel happy now) changes.	Focus on common disorders to limit the volume of literature, but maintain coverage of likely links between environmental science and mental health research.

First pass on title – papers are identified as potentially relevant or rejected based on inclusion criteria. Reason for rejection recorded in spreadsheet:

Paper ID code	Title	Potentially relevant	Rejected: Mental health scope	Rejected: Environmental Science scope	Rejected: Review paper	Etc…

Second pass on abstract is carried out on only those identified as potentially relevant by title. Papers are accepted or rejected based on inclusion criteria. If rejected reason recorded. If accepted paper classified as physical, biological and/or chemical (and potentially pollution, flooding, biodiversity etc). All papers that are accepted will go on to have full text analysed.

Paper ID code	Accepted?	Physical?	Biological?	Chemical?	Rejected: Mental health scope	Rejected: Environmental Science scope	Etc…

To check consistency 10% of abstracts screened for acceptance/rejection and classification by a second person. Classifications compared and should meet 90% match.

Amendment to protocol: Given the high agreement (96%) between researchers at the title screening stage it was decided that further consistency checks at the abstract stages was not necessary.

Because of the large number of papers remaining in the sample following abstract screening, and the large number of recent reviews identified on climate change, flooding, air pollution and greenspace it was decided to remove these papers from the full text review, and instead draw from the review papers for this topic.

Extraction strategy

All papers identified as relevant by their abstract will have full text analysed. They will be grouped into physical, biological or chemical environmental science, and each group of papers assessed by a different pair of researchers (chemical ZZ and KI; biological KC and AE, physical MC and MR). Where papers related to multiple areas of environmental science (e.g. flooding (physical) and water pollution (chemical) they will be divided among researchers separately to avoid duplication of effort. Data extraction is concerned primarily with results, rather than interpretation by the authors.

Amendment to protocol: Due to researcher availability data extraction was carried out by MR, KI, KC and MC only. Papers were split evenly among researchers, with qualitative papers split between KI and MC only. Papers were not grouped by environmental science area.

Each line on the extraction spreadsheet will refer to an individual link between environmental science and mental health outcome. For example, if a study looked at flooding and water pollution in relation to depression and anxiety four lines would be added:

- Flooding to depression
- Flooding to anxiety
- Water pollution to depression
- Water pollution to anxiety

This will mean some duplication of data (e.g. in the study type column), but is important for later sorting the data

Data extracted into extraction spreadsheet including:

Paper ID code – author and date, matched to abstract spreadsheet
Study type – Quantitative/qualitative/mixed
Study design – BACI (Before After Control Impact), correlation etc
Population – As described (e.g. over 65's living in tower blocks)
Sample size
How sample recruited

Amendment to protocol: Is the sample intended to be representative?

Geographical Location
Disciplinary field – Is the research approached from environmental science or mental health starting point, both, or unclear?
Methods type - Lab/Field/Epidemiological/secondary
Environmental Science methods – e.g. Water framework directive classification, water testing
Environmental Science measures – e.g. water pollution levels, biodiversity
Mental health methods – e.g. interviews, psychometric scales, population level data
Mental health measures – e.g. wellbeing metrics, prescription rates, diagnosis
Additional variables included in the model – e.g. age, gender, occupation

Amendment to protocol: Removal of: What are they calling the pathway – e.g. association, causal, associative etc, because this was decided to be more specific to discipline than study type.

Analysis – e.g. GLM, thematic analysis
Main results –. Positive, negative, null.
Results summary - Limiting to results presented, not author interpretation
Future research (methods) - Methodological suggestions for future research. As found either in 'Future research' section, 'conclusions' section or ctrl+F for the phrase 'future research'.
Future research (environmental science) - Environmental science suggestions for future research. E.g. water pollution in total rather than specific measure. As found either in 'Future research' section, 'conclusions' section or ctrl+F for the phrase 'future research'.
Future research (mental health) - Mental health suggestions for future research. E.g. self reported rather than diagnoses. As found either in 'Future research' section, 'conclusions' section or ctrl+F for the phrase 'future research'.
Future research (questions) - Suggestions for future research questions. As found either in 'Future research' section, 'conclusions' section or ctrl+F for the phrase 'future research'.
Extent of interaction (Huutoniemi et al 2010):

- Composite multidisciplinary - Expertise in different fields combined, but research is still modular and still framed within a single discipline. For us this might be the inclusion of tree cover as a variable in a model linking access to healthcare with depression diagnoses.

- Empirical interdisciplinarity - Integration of empirical data from multiple disciplines to answer a question around the relationship between both disciplines. This could

be correlation between air pollution and wellbeing. Most of our studies probably fit here.

- Methodological interdisciplinarity - Combining and integration of methods to suit interdisciplinary nature of question. This could be monitoring of brain activity or wellbeing measures related to stress while experiencing simulation of flooding event.

- Theoretical interdisciplinarity - Synthesis of concepts, models or theories from multiple disciplines, forming interdisciplinary theory. This could include model integrating social, environmental, health and economic risk factors in schizophrenia, investigating mediating factors and pathways which interact to lead to physiological responses and development of the disorder.

Details of evidence synthesis

Details of volume and characteristics of evidence, including:

Field, study types and designs
Populations and geographic spread
Methods and measures used
Extent of interaction
Outcomes reported – identifying consistent (+ve, -ve, 0) or mixed evidence

Narrative synthesis of outcomes reported, organised by environmental science type or mental health. Alongside the summary, identify where strengths in collaboration are evident, and where evidence is missing. Consider implications for future research and funding needs.

Outline of conflicts of interest and sources of support

Funded by Natural Environment Research Council via Valuing Nature Programme

References for protocol

Arksey, H. and O'Malley, L., 2005. Scoping studies: towards a methodological framework. *International journal of social research methodology*, *8*(1), pp.19-32.

Barton, H. and Grant, M., 2006. A health map for the local human habitat. *Journal of the Royal Society for the Promotion of Health*, *126*(6).

Collins, A., Coughlin, D., Miller, J. and Kirk, S., 2015. The production of quick scoping reviews and rapid evidence assessments: a how to guide.

Huutoniemi K, Klein JT, Bruun H, Hukkinen J. Analyzing interdisciplinarity: Typology and indicators. *Research policy*. 2010 Feb 1;39(1):79-88.

Munn, Z., Peters, M.D., Stern, C., Tufanaru, C., McArthur, A. and Aromataris, E., 2018. Systematic review or scoping review? Guidance for authors when choosing between a systematic or scoping review approach. *BMC medical research methodology*, *18*(1), p.143.

Sucharew, H. and Macaluso, M., 2019. Methods for Research Evidence Synthesis: The Scoping Review Approach. *Journal of hospital medicine*, *14*(7), pp.416-8.

Timeline

	June			July				Aug					Sep				Oct				Nov					Dec				Jan				Feb			
	15	22	29	6	13	20	27	3	10	17	24	31	7	14	21	28	5	12	19	26	2	9	16	23	30	7	14	21	28	4	11	18	25	1	8	15	22
Protocol – First draft																																					
Protocol– Team feedback																																					
Protocol – Second draft																																					
Protocol – expert feedback																																					
Workshops																																					
Protocol finalised																																					
Evidence search																																					
Screen results																																					
Extract evidence																																					
Synthesis evidence																																					
Draft report, case studies and recommendations																																					
Final report, case studies and recommendations																																					
Manuscript																																					

Amended timeline – accounting for delay in data extraction and additional report for NERC. Amended times in orange.

	June			July				Aug					Sep				Oct				Nov					Dec				Jan				Feb				March			
	15	22	29	6	13	20	27	3	10	17	24	31	7	14	21	28	5	12	19	26	2	9	16	23	30	7	14	21	28	4	11	18	25	1	8	15	22	1	8	15	22
Protocol – First draft																																									
Protocol– Team feedback																																									
Protocol – Second draft																																									
Protocol – expert feedback																																									
Workshops																																									
Protocol finalised																																									
Evidence search																																									
Screen results																																									
Extract evidence																																									
Interim report																																									
Synthesis evidence																																									
Draft report, case studies and recommendations																																									
Final report, case studies and recommendations																																									
Manuscript																																									
